# Autoantibodies Targeting Intracellular and Extracellular Proteins in Autoimmunity

**DOI:** 10.3389/fimmu.2021.548469

**Published:** 2021-03-08

**Authors:** Peter D. Burbelo, Michael J. Iadarola, Jason M. Keller, Blake M. Warner

**Affiliations:** ^1^ National Institute of Dental and Craniofacial Research, National Institutes of Health, Bethesda, MD, United States; ^2^ Department of Perioperative Medicine, Clinical Center, National Institutes of Health, Bethesda, MD, United States; ^3^ Salivary Disorders Unit, National Institute of Dental and Craniofacial Research, National Institutes of Health, Bethesda, MD, United States

**Keywords:** autoantibodies, autoimmune, treatment, onset, autoantigen

## Abstract

Detecting autoantibodies provides foundational information for the diagnosis of most autoimmune diseases. An important pathophysiological distinction is whether autoantibodies are directed against extracellular or intracellular proteins. Autoantibodies targeting extracellular domains of proteins, such as membrane receptors, channels or secreted molecules are often directly pathogenic, whereby autoantibody binding to the autoantigen disrupts the normal function of a critical protein or pathway, and/or triggers antibody-dependent cell surface complement killing. By comparison, autoantibodies directed against intracellular proteins are recognized as useful diagnostic biomarkers of abnormal autoimmune activity, but the link between antigenicity and pathogenicity is less straightforward. Because intracellular autoantigens are generally inaccessible to autoantibody binding, for the most part, they do not directly contribute to pathogenesis. In a few diseases, autoantibodies to intracellular targets cause damage indirectly by immune complex formation, immune activation, and other processes. In this review, the general features of and differences between autoimmune diseases segregated on the basis of intracellular or extracellular autoantigens are explored using over twenty examples. Expression profiles of autoantigens in relation to the tissues targeted by autoimmune disease and the temporal appearance of autoantibodies before clinical diagnosis often correlate with whether the respective autoantibodies mostly recognize either intracellular or extracellular autoantigens. In addition, current therapeutic strategies are discussed from this vantage point. One drug, rituximab, depletes CD20+ B-cells and is highly effective for autoimmune disorders associated with autoantibodies against extracellular autoantigens. In contrast, diseases associated with autoantibodies directed predominately against intracellular autoantigens show much more complex immune cell involvement, such as T-cell mediated tissue damage, and require different strategies for optimal therapeutic benefit. Understanding the clinical ramifications of autoimmunity derived by autoantibodies against either intracellular or extracellular autoantigens, or a spectrum of both, has practical implications for guiding drug development, generating monitoring tools, stratification of patient interventions, and designing trials based on predictive autoantibody profiles for autoimmune diseases.

## Introduction

The production of autoantibodies against self-proteins, called autoantigens, is an abnormal process characteristic of most autoimmune diseases. Autoantibody immunoreactivity in patient blood or CSF provides key diagnostic information when autoimmune disease is suspected. The spectrum of autoantibodies is often clinically informative for a given autoimmune disease. Some autoimmune diseases harbor autoantibodies against only one or a few target autoantigens, but in other conditions autoantibodies against multiple targets may co-exist. Among the seventy most common autoimmune diseases, approximately 100 out of the estimated 20,000 human proteins encoded by the genome comprise the most common antigenic targets (1). However, an increasing number of autoantibodies have been discovered in rare disorders suggesting additional diseases are likely to exhibit autoantibody-associated autoimmunity.

The treatment of many autoimmune diseases remains sub-optimal due to varying degrees of efficacy and the side-effects of available interventions (2). Advances in autoimmune disease therapeutics will require disease-specific information including identification of immune cells and underlying signaling pathways involved, the mechanisms governing loss of tolerance, and how autoantibodies participate in pathogenesis. To aid in this process, we have focused on conceptually defining autoimmune diseases based on whether the autoantibodies in a particular disorder target extracellular or intracellular proteins or a more complex mixture of both. Intracellular autoantigens are generally inaccessible to binding by autoantibodies and instead represent autoimmune biomarkers of abnormal immune cell activity. Conversely, extracellular antigenic proteins are readily accessible to autoantibodies whose targets include secreted proteins, cell surface channels, and receptors. Antibodies against extracellular proteins can directly cause disease by altering protein function or abundance and/or by recruiting complement-mediated cell killing. In this review, we explore the potential for classification of autoimmune diseases based on whether they have autoantibodies predominantly against intracellular or extracellular targets. These two distinct sites of autoantigen localization are described in the context of tissue expression, temporal appearance of autoantibodies in relation to disease diagnosis, and how this information provides a rational basis for treatment choices.

### Autoimmune Diseases Enriched in Autoantibodies Against Intracellular Proteins

Some of the most common autoimmune diseases demonstrate a preponderance of autoantibodies directed against intracellular targets including structural proteins, enzymes, splicing machinery, RNA-binding proteins, and RNA polymerases (3). Although it is well-recognized that T-cells play a central role in the destruction of the corresponding cells and tissue in autoimmune diseases showing autoantibodies against intracellular proteins additional mechanisms have been proposed to explain why these proteins become targets of B-cell responses including release from dying cells, ineffective clearance of apoptotic debris, protein modification during inflammatory responses, and molecular mimicry (4). In this section, we discuss several major autoimmune diseases characterized mainly by autoantibodies to intracellular proteins and describe the tissue expression patterns of their target autoantigens in relationship to the autoimmune process and its pathological manifestations.

**Figure 1:**
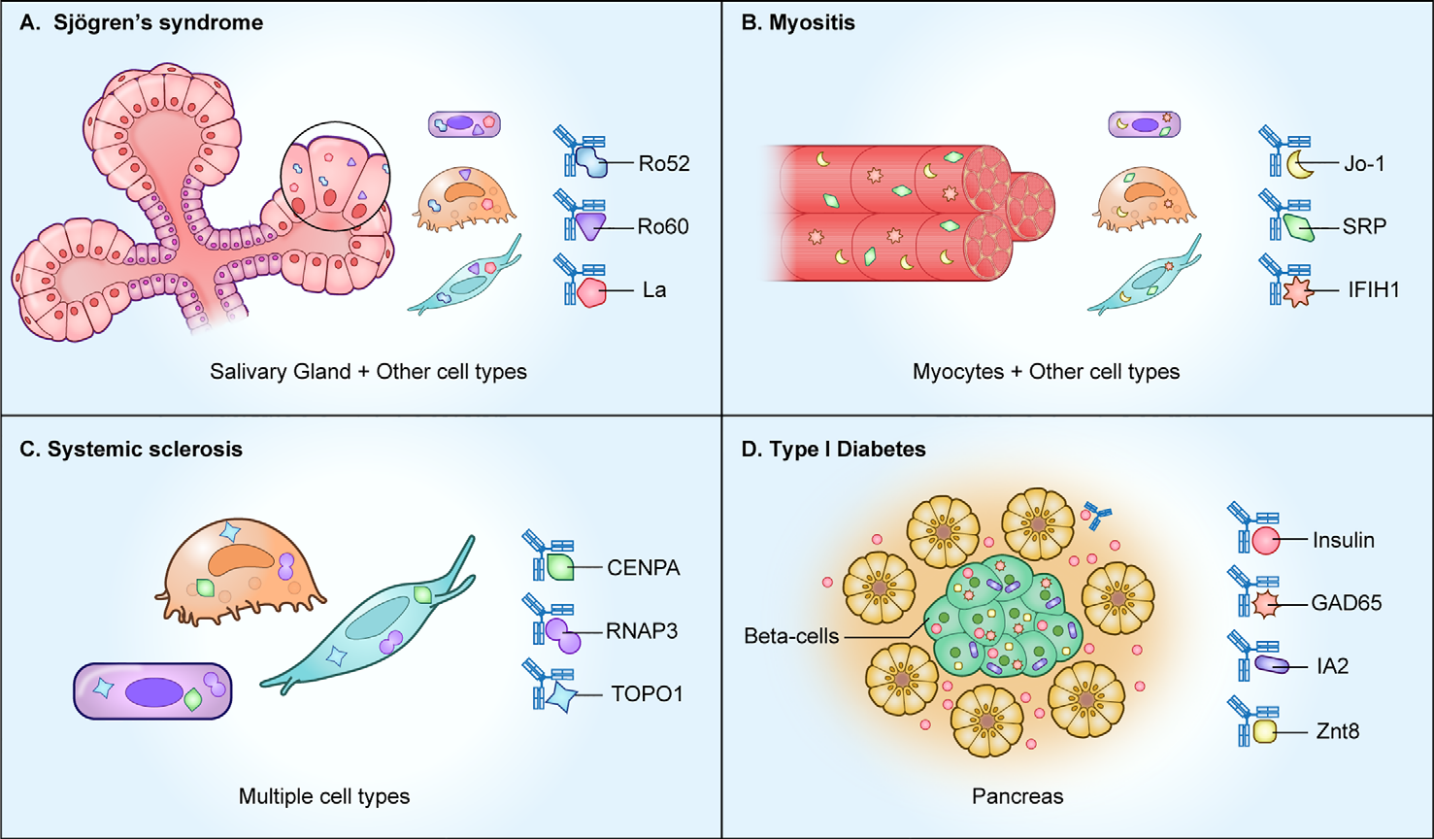
Autoimmune diseases with autoantibodies directed against intracellular proteins. As shown, several autoimmune diseases including **(A)** Sjögren’s syndrome, **(B)** myositis, and **(C)** systemic sclerosis, harbor autoantibodies against ubiquitously expressed intracellular proteins. However, in **(D)** type I diabetes, the intracellular autoantigens represent beta cell-specific proteins derived from the pancreas.

Autoantibodies against intracellular proteins are important disease biomarkers in a number of rheumatological diseases including Sjögren’s syndrome, systemic lupus erythematosus, systemic sclerosis, and myositis. In Sjögren’s syndrome, an autoimmune disease defined by sicca symptoms of oral and ocular dryness, the major autoantibodies are against SSA and SSB. SSA is comprised of two different autoantigenic proteins, Ro52 (*TRIM21)* and Ro60 (*Trove2)*, and SSB autoantibodies recognize a single protein, La (*SSB*) (**Figure 1A**). Despite the importance of those three autoantigens for diagnosing an underlying autoimmune basis for Sjögren’s syndrome, they are ubiquitously expressed, rather than confined only to salivary glands, thus making a causal relationship to sicca symptoms difficult to establish (3). Both Ro60 and La are intracellular RNA-binding proteins, but Ro52 acts differently, as an important immunoglobulin receptor inside cells that mediates ubiquitylation-dependent destruction and neutralization of internalized immunoglobulin-pathogen complexes (4). Based on the biological function of Ro52 in pathogen clearance, one possible abnormal mechanism explaining its antigenicity in subjects with Sjögren’s syndrome and other autoimmune diseases is that the entire protein complex of Ro52, immunoglobulins, and infectious agents such as virus may be recognized as “foreign” (5).

Systemic lupus erythematosus (SLE) is characterized by immune activation and widespread tissue destruction (6). A common feature of SLE is the high prevalence of autoantibodies to several intracellular proteins, as well as against cellular DNA. In addition to autoantibodies against Ro52, Ro60 and La proteins, RNA-binding proteins including RNP-A (*SNRPA1*), U1-70K (***SNRNP70*),** and Sm-D3 (*SNRPD3*), are also important diagnostic autoantigens. It is important to point out that these autoantibodies against intracellular targets are not directly pathogenic *via* their autoantigen binding, but they can still contribute to disease by participating in immune complex formation, complement activation, and immune activation (7, 8). Exactly why these various RNA-binding proteins are autoantigenic or what causes loss of tolerance to them is unknown. Autoantibody profiling reveals that most SLE patients can be segregated into one of two autoantibody clusters: those with Ro52, Ro60, and La as targets, or those who are enriched for Sm-D3, U1-70k, and RNP-A autoantibodies (9). Numerous clinical phenotyping efforts have found that autoantibodies to certain intracellular autoantigens correlate with specific SLE symptoms (10–12). For example, the presence of RNP-A autoantibodies is associated with patients having Raynaud’s skin symptoms. Since none of these intracellular RNA-binding proteins are accessible to autoantibody binding, one possible explanation for their association with certain symptoms is that these autoantibodies may converge on common protein synthesis pathways involved in disease pathogenesis, and upon upregulation and release, these RNA-binding proteins become autoantibody-associated biomarkers.

Myositis represents a diverse spectrum of disease subtypes involving autoimmune-mediated muscle inflammation and subsequent muscle tissue destruction (13). All known myositis autoantigens that are targets of autoantibodies are intracellular proteins (**Figure 1B**). By profiling the autoantibody response against a panel of myositis-associated autoantigens, it is possible to segregate the disease into four subtypes: anti-synthetase syndrome, dermatomyositis, inclusion body myositis, or immune-mediated necrotizing myopathy (14). In anti-synthase syndrome, the major autoantigens are involved in tRNA synthesis and include Jo1, an enzyme responsible for histidyl-tRNA synthesis (*HARS*), PL7, a threonyl tRNA synthetase (*TARS*), and PL-12 alanyl-tRNA synthetase (*ARS*). In dermatomyositis, autoantibodies against Mi2 histone acetylase, the tRNA synthetase proteins and anti-TIF-γ are often found. Dermatomyositis patients with interstitial lung disease often harbor autoantibodies against an intracellular RNA sensor protein, MDA-5 (*IFIH1*), but the mechanism behind the association of autoantibodies with lung disease is not known. Lastly, in necrotizing myositis, autoantibodies directed against the intracellular signal recognition protein (SRP) complex and in some rare cases against 3-hydroxy-3-methylglutaryl-coenzyme A reductase (*HMGCR*) are present. The association and pathways involved in generating autoantibodies against these diverse intracellular autoantigens in myositis remains unresolved.

The autoimmune manifestations in systemic sclerosis (scleroderma) are characterized by vascular dysfunction, inflammation, and fibrotic structural changes in the skin and internal organs (15). As shown in **Figure 1C**, patients exhibit autoantibodies against a variety of intracellular proteins that include Ro52, Ro60, topoisomerase 1 (top1), centromere proteins (*CENPA* and *CENPB*), PM/SCL (*EXOSC9* and *EXOSC107*), and RNA polymerase 3 complex (*POLR3A* and *POLR3K*) (16). Autoantibody-based diagnosis in systemic sclerosis patients requires a large panel of fifteen autoantigens for high diagnostic sensitivity to classify most systemic sclerosis patients into one of five clusters (17). Autoantibody-mediated pathways targeting extracellular proteins have been explored as possible drivers of fibroblast activation, but to date none have been discovered.

Type I diabetes (T1D) is an autoimmune disease commonly found in children involving T-cell mediated immune destruction of the insulin-producing beta cells in the pancreas (18). Autoantibodies against one extracellular and several intracellular proteins are typically found in T1D and represent important biomarkers for the disease (**Figure 1D**). Autoantibodies against the secreted peptide hormone insulin (*INS*), an extracellular target, are one of the early indicators of prediabetic islet cell autoimmunity in T1D (19). Despite the accessibility of circulating insulin to serum autoantibodies, anti-insulin autoantibodies are not pathogenic because they do not cause the destruction of the insulin-producing beta-cells. The intracellular autoantigen IA-2 (*PTRN*) is a receptor type tyrosine-protein phosphatase localized to the membrane of dense core vesicles and highly expressed in the brain and pancreas. The region of IA2 directed toward the vesicle lumen is immunodominant, and this intracellular tail region is used to measure autoantibodies in most studies (19). Two more intracellular proteins, glutamate decarboxylase/GAD65 (*GAD2*) and Znt8 (*SLC30A8*), are also targets of autoantibodies in T1D. GAD65 is an enzyme responsible for synthesis of the neurotransmitter gamma-aminobutyric acid (GABA). Autoantibodies against GAD65 are not specific to T1D and can be found in several central nervous system autoimmune diseases, including Stiff-person syndrome and autoimmune-mediated encephalitis (20). Znt8 is an abundantly expressed zinc transporter protein found on insulin secretory granules of pancreatic beta cells. Besides beta cell-specific proteins, autoantibodies against the ubiquitously-expressed proteins tetraspanin-7 are found in T1D (*TSPAN7*) (21). Protein array technologies have also identified the intracellular peptidylprolyl isomerase like 2 (*PPIL2*) and DNA mismatch repair protein Mlh1 (*MSH1*), albeit the presence of these autoantibodies only occurs in a small subset (<8%) of T1D subjects (22, 23). Understanding the loss of B-cell tolerance to these rarer autoantigens may provide insight into patient subsets, autoantibody spreading, and/or mechanisms involved in T1D autoimmunity.

In addition to proteins that are strictly intracellular, there are some autoantigens that are transiently expressed on the cell surface, thereby becoming accessible to autoantibody binding. In systemic vasculitis, the intracellular target autoantigens are proteinase-3 (PR3) and myeloperoxidase (MPO), which have signal peptides that allow their association with and storage in secretory vesicles. In patients with vasculitis activated neutrophils, PR3 and MPO proteins traffic to the plasma membrane surface (24, 25), making these normally intracellular primary granule enzymes accessible to autoantibody binding. PR3 and MPO autoantibody binding can activate neutrophils by engaging Fcγ receptors (26). Passive transfer of MPO autoantibodies into recipient mice cause glomerulonephritis and vasculitis and provides further proof that these autoantibodies are pathogenic (27).

With the exception of T1D, where autoantigens are generated against highly expressed proteins of the insulin-producing beta cells of the pancreas, the relationships between autoantigenic proteins and target tissues in the other autoimmune disease examples remains poorly understood. For example, it is unclear how autoantibodies against Ro52 and Ro60 proteins in Sjögren’s syndrome act as biomarkers for salivary gland dysfunction and why the ubiquitous t-RNA synthetases are targets of autoantibodies in myositis. Understanding the mechanisms involved in the loss of tolerance against these and other autoantigens would shed light on how autoimmunity develops and what are the etiological triggers for these autoimmune diseases.

### Autoantibody Diseases Harboring Autoantibodies to Extracellular Targets

**Figure 2:**
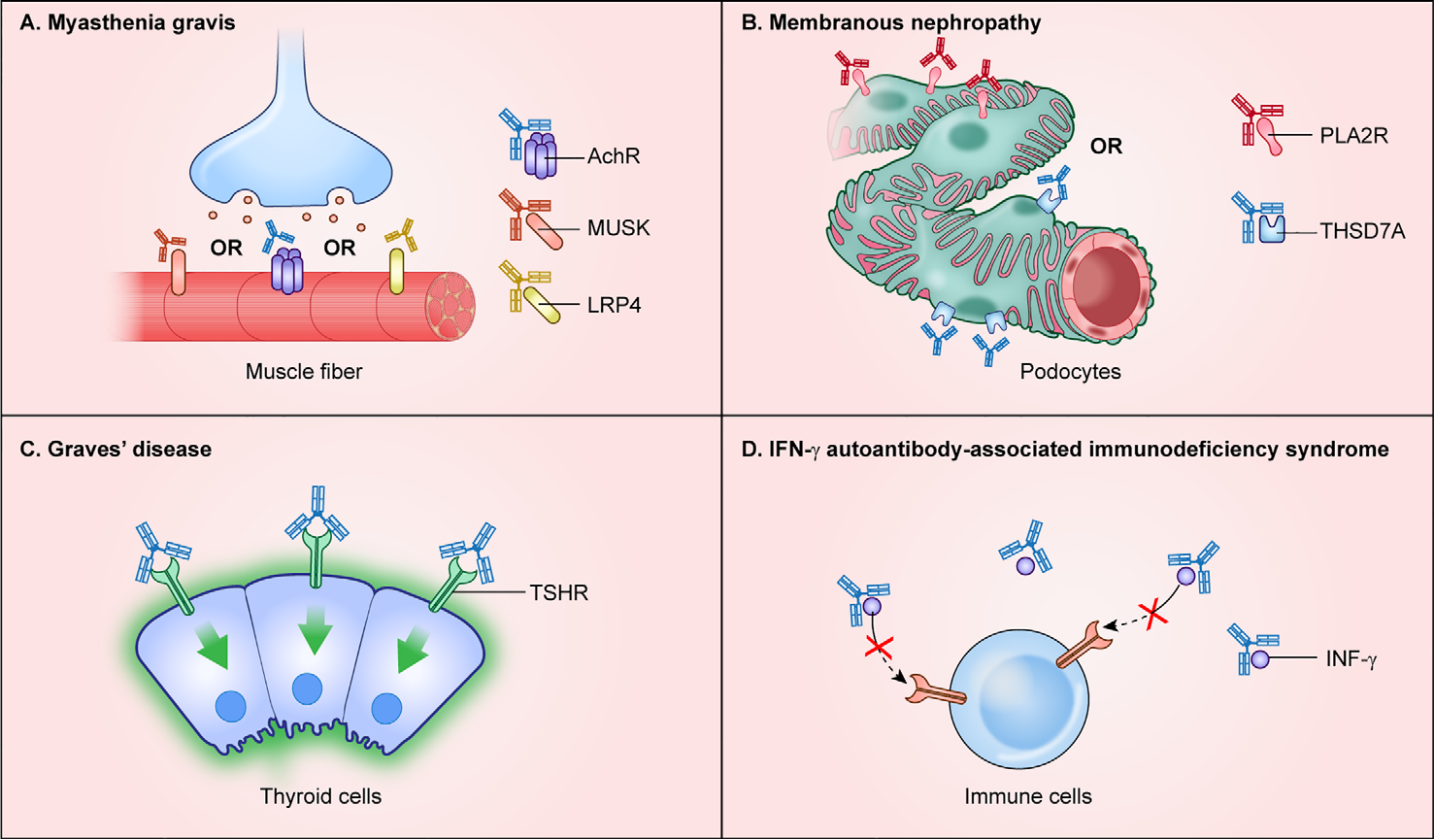
Autoimmune diseases with autoantibodies directed against extracellular protein targets. Autoantibodies targeting extracellular proteins directly cause disease pathogenesis and are found in autoimmune disease including **(A)** myasthenia gravis, **(B)** membranous nephropathy, **(C)** Graves’ disease, and **(D)** interferon-γ autoantibody immunodeficiency syndrome.

Autoantibodies against extracellular proteins can directly cause a variety of autoimmune diseases. This direct pathogenicity is caused by binding of the autoantibody to the extracellular protein thereby disrupting normal function of a critical protein or pathway, and/or by triggering antibody-dependent cell surface complement killing. In order to classify an autoantibody against an extracellular target as pathogenic, it needs to fulfill several criteria: 1) the specific autoantibody is strongly associated with the relevant clinical presentation of the disease and absent in healthy individuals or other diseases, 2) the autoantigen is specifically localized to the diseased tissue, and 3) the autoantibody levels correlate with disease activity. Validating the pathogenicity of autoantibodies often involves animal models, whereby passive transfer of patient autoantibodies or antigen-induced immunization can recapitulate clinical features of the disease. An in-depth discussion of mechanisms underlying autoantibody-induced pathology can be found in a recent review (28). Here, we provide examples of several major autoimmune diseases harboring pathogenic autoantibodies to extracellular autoantigenic targets and describe how many of these autoantigen targets are cell/tissue-specific and fulfill the criteria for pathogenicity (**Figure 2**).

Myasthenia gravis is an autoimmune disease of muscle harboring pathogenic autoantibodies that interfere with cholinergic receptors and other proteins at the neuromuscular junction. The autoantibodies found in myasthenia gravis cause progressive skeletal muscle weakness (29). The major autoantibody target, found in approximately 85% of myasthenia gravis patients, is the extracellular N-terminal region of the alpha 1 subunit of the nicotinic acetylcholine receptor/nAChR (*CHRNA1*), which is highly enriched in skeletal muscle (**Figure 2A**). Autoantibodies against two additional targets, the muscle-associated cell surface tyrosine kinase (*MUSK*) and the low-density lipoprotein receptor-related protein (*LRP4*), are less common and found in about 1–10% and 1–3% of cases, respectively (29) (**Figure 2A**). Patients seropositive for either nAChR or LRP4 autoantibodies show classic myasthenic symptoms, yet interestingly patients with MuSK autoantibodies show more bulbar and cranial involvement, less muscle extremity involvement, and a high occurrence of respiratory problems. As illustrated in myasthenia gravis patients, and in most patients with autoantibodies to an extracellular target, the humoral response is directed against only one target protein, which typically drives pathogenesis.

Autoimmune diseases of the central nervous system also involve autoantibodies to extracellular proteins found in neurons or glia (30). For example, autoantibodies targeting the aquaporin-4 (*AQP4*) channel, which is enriched on the surface of astroglial cells and involved in maintaining integrity of the blood brain barrier, cause neuromyelitis optica (NMO/Devic’s disease). Similarly, autoantibodies against myelin oligodendrocyte glycoprotein (MOG) cause autoimmune demyelination disease in NMO. Autoantibodies targeting subunits of the N-methyl-D-aspartate receptor/NMDAR (e.g., *GRIN1*) on the surface of neurons cause encephalitis and other neurological problems (31). Additional autoimmune neurological diseases targeted by pathogenic autoantibodies involve alpha-amino-3-hydroxy-5methyl-4-isoxazolepropionic receptor/AMPAR (*GRIA1* and *GRIA2*) and channel scaffold proteins such as the secreted leucine rich glioma inactivated 1 protein (*LGI1*) and the neurexin family protein contactin associated protein 2 (*CNTNAP2*) (30). Deleterious effects in the central nervous system often involve interference with the normal function of critical ion channels.

Several autoimmune diseases of the kidney are driven by autoantibodies to extracellular proteins. Anti-glomerular basement membrane disease (Goodpasture’s syndrome), designated anti-GBM disease, is caused by autoantibodies against the collagen IV-alpha 3 chain (*COL4A3*) whose expression is enriched in lung and kidney (32). In anti-GBM disease, autoantibodies against collagen IV cause complement activation and leukocyte infiltration that damages the basement membrane lining the capillaries in the glomeruli of the kidney. A different autoimmune condition, membranous nephropathy, exhibits focal autoantibody deposits in the kidney sub-epithelial layer of the glomerular basement membrane adjacent to podocyte foot processes (33). Autoantibodies in membranous nephropathy are directed against at least two podocyte-specific membrane proteins with extracellularly exposed regions including phospholipase A2 receptor (*PLA2R*) (34) and thrombospondin type-1 domain-containing 7A (*THSD7A*) (35) (**Figure 2B**). The mRNAs and proteins for PLA2R and THSD7A show some of the highest expression levels in the kidney. PLA2R autoantibodies are the most common cause of membranous nephropathy and can be used diagnostically or for monitoring responses to therapy, as well as for detecting relapse (36).

Autoantibodies to extracellular target proteins are typically assumed to cause a corresponding loss of function. However, in Graves’ thyroiditis, binding of autoantibodies to the thyroid hormone stimulating receptor (*THSR*) found on follicular thyroid cells has an agonist-like activity that over-activates downstream signaling and results in high levels of circulating thyroid hormones (37) (**Figure 2C**). Clinical symptoms include hyperthyroidism, ophthalmopathy, and dermopathy (37). Besides the thyroid-stimulating hormone receptor, autoantibodies are also directed to intracellular autoantigens including thyroid peroxidase (*TPO*), which is involved in thyroxine biosynthesis, and to the secreted thyroid hormone binding protein, thyroglobulin (*TG*). Although thyroid-stimulating hormone receptor autoantibodies are well-established as the cause of hyperthyroidism in Graves’ disease, less is known about the mechanisms underlying other features such as ophthalmopathy or dermopathy.

Autoantibodies against circulating hormones, growth factors, and cytokines cause a variety of autoimmune-mediated diseases. Cytokines are particularly important because they function as key regulators of the immune system by playing critical roles in the maturation of immune cells and orchestrating responses to pathogens. Several acquired autoimmune immunodeficiencies are caused by anti-cytokine autoantibodies (38). One anti-cytokine autoimmune disease is pulmonary alveolar proteinosis caused by autoantibodies against GMCSF (*CSF2*) (39). Autoantibodies sequester GMCSF and block its signaling, thereby preventing downstream production and maturation of macrophages in the lung, thus leading to excessive accumulation of surfactant and other lipoproteins in the lower respiratory tract. While the lung is the most vulnerable organ, a second clinical phenotype found in patients with anti-GMCSF autoantibodies are opportunistic infections by microbes such as *Cryptococcus*, *Nocardia*, and *Histoplasma*, which are caused by defective phagocyte function (40, 41). Another acquired anti-cytokine immunodeficiency syndrome is caused by autoantibodies against interferon-γ (*IFNG*), in which patients develop severe mycobacterial infection (42). The IFN-γ autoantibodies detected in these patients are mainly of the IgG4 isotype and bind circulating IFN-γ, interfering with its normal signaling activity (**Figure 2D**). Consistent with this sequestration mechanism, serum autoantibodies harvested from patients were capable of neutralizing *in vitro* signaling activity downstream of the IFN-γ receptor as demonstrated by blockade of STAT1 phosphorylation (42). These and other examples of anti-cytokine autoimmune diseases highlight how vulnerabilities to specific infectious agents is driven by loss of function of specific cytokines responsible for proper immune cell signaling.

It is important to point out that some individuals with autoantibodies against extracellular proteins also occasionally have additional autoantibodies directed against intracellular proteins, but that the defining pathology is caused by autoantibodies against the extracellular autoantigen. Recognizing that autoantibodies against a specific target protein drive the clinical features of a disease is an important aspect to consider with regard to treatment. One benefit to monitoring serum levels of pathogenic autoantibodies is the ability to directly track responses to therapy, where the reduction or disappearance of circulating autoantibodies coincides with cure or remission.

### Autoimmune Diseases Harboring Autoantibodies Against Extracellular Proteins Can Mimic Genetic Diseases for the Same Target Protein or Pathway

One interesting feature of pathogenic autoantibody diseases associated with extracellular autoantigens is that they frequently share clinical phenotypes with genetic mutations in the corresponding protein target or pathway (**Table 1**). This relationship between autoimmune-mediated and a corresponding inherited genetic disease in the same protein is consistent with the loss-of-function phenotype induced by most acquired pathogenic autoantibodies. For example, in congenital forms of myasthenia gravis, patients possess mutations either in the alpha1 subunit of the acetylcholine receptor (*CHRNA1*), *LRP4*, or *MUSK* genes, all known targets of autoantibodies causing autoimmune forms of myasthenia gravis (43). In anti-GBM autoimmune disease, there are autoantibodies to the collagen IV-alpha 3 chain and in the genetic disease Alport syndrome, glomerulonephritis, and end-stage kidney disease are caused by mutations in the collagen IV-alpha 3 chain (44). Mutations in *GPIHBP1*, encoding a protein involved in blood lipid transport, causes hyperlipidemia (46) with clinical features mimicking autoimmune hyperlipidemia caused by inactivating autoantibodies against GPIHBP1 (46). In some cases, the genetic defect occurs in the receptor rather than the ligand, resulting in the same phenotype. For example, pulmonary alveolar proteinosis (PAP) patients have autoantibodies against the soluble GMCSF cytokine preventing the normal development of macrophages in the lung, but the genetic forms of PAP have mutations in the membrane-bound GMCSF receptor (*CSF2RA*) (51). Similarly, patients with mutations in the interferon-gamma receptor (*IFNGR*) (52) exhibit clinical features resembling patients with neutralizing autoantibodies against the cognate ligand, interferon-gamma (42), resulting in unusual opportunistic non-mycobacterial infections. While diseases caused by gene mutations are inherited as life-long conditions and are difficult to treat, the analogous autoimmune diseases are acquired and often highly treatable with immune therapies.

**Table 1:** Pathogenic autoantibody-mediated diseases mimic genetic diseases.

Gene or protein	Genetic mutation phenotype	Autoimmune phenotype
**CHRNA1 (AchR1)**	Congenital myasthenia gravis (43)	Myasthenia gravis (29)
**MUSK**	Congenital myasthenia gravis (43)	Myasthenia gravis (29)
**COL4A3**	Alport syndrome (44)	Anti-GBM disease (32)
**LRP4**	Congenital myasthenia gravis (43)	Myasthenia gravis (29)
**GPIHBP1**	Hyperlipidemia (45)	Hyperlipidemia (46)
**GluR1 (NMDA)**	Epilepsy (47)	Epilepsy and encephalitis (31)
**ADAMTS13**	Congenital thrombotic thrombocytopenic purpura (48)	Autoimmune thrombotic thrombocytopenic purpura (48)
**FGF23**	Familial hyperphosphatemic tumoral calcinosis (49)	Autoimmune hyperphosphatemic tumoral calcinosis (50)
**CSF2RA (receptor) or GMCSF (ligand)**	*CSFR2* mutations cause hereditary alveolar proteinosis (51)	GMSF autoantibodies cause alveolar proteinosis (39)
**IFNGR (receptor) or IFN-γ (ligand)**	*IFNRG* mutations cause opportunistic mycobacterial infections (52)	IFN-γ autoantibodies cause opportunistic mycobacterial infections (42)

In contrast, there is little or no evidence that mutations in genes encoding intracellular autoantigens cause similar diseases. For example, autoantibodies against the intracellular autoantigen MDA5, encoded by the *IFIH1* gene, are found in myositis-associated lung disease, but mutations in the *IFIH1* gene cause an unrelated disease characterized by a spectrum of neuro-immunological features (53). Similarly, mutations in HARS, encoding the intracellular Jo-1 myositis autoantigen, do not affect muscle tissue but cause a genetic form of inherited Charcot-Marie-Tooth type 2 peripheral neuropathy (54).

Based on the observation that a variety of genetic diseases involve mutations in extracellular receptors and secreted molecules, we speculate that there are likely more unrecognized autoimmune conditions involving autoantibodies to extracellular targets. This may be particularly applicable to patients with unknown disease etiology where whole exome sequencing has not identified coding mutations, thereby implicating alternative mechanisms. One recent example was the identification of autoimmune hyperphosphatemic tumoral calcinosis in a child exhibiting paradoxically high, unexplained levels of FGF23 cytokine, but showing resistance to FGF23 signaling (50). Targeted genetic analysis and whole exome sequencing of the affected child did not reveal mutations in any known or candidate genes. However, serological testing revealed that the child was robustly seropositive for autoantibodies against FGF23, and these anti-FGF23 autoantibodies were able to interfere with FGF23 signaling. This case study highlights how pathogenic autoantibodies directed against extracellular proteins associated with known genetic diseases can cause a similar clinical phenotype. These findings provide the rationale for evaluating autoantibodies against their corresponding extracellular targets as alternate mechanisms of disease pathogenesis.

### Autoantibody Appearance Before Autoimmune Disease Diagnosis

**Figure 3:**
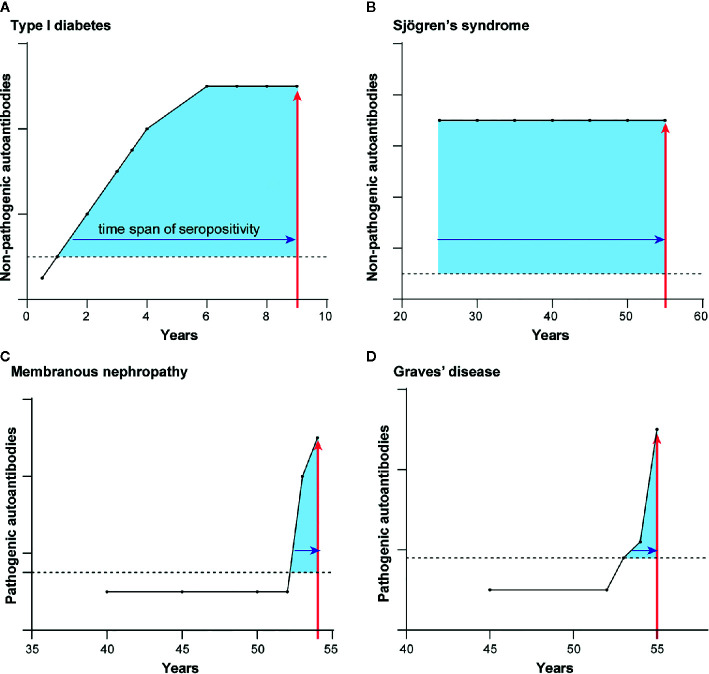
Autoantibody appearance before autoimmune diagnosis differ for the two types of autoimmune diseases. Representative illustrations for the typical time course of prediagnostic autoantibodies before the diagnosis of diseases harboring autoantibodies to intracellular proteins **(A, B)** and extracellular proteins **(C, D)**. Shown are the longitudinal appearance of autoantibodies in **(A)** TD1, **(B)** Sjögren’s syndrome, **(C)** membranous nephropathy, and **(D)** Graves’ disease. Time of autoimmune disease diagnosis is shown by the vertical red line arrow. The length of time of autoantibody seropositivity before the time of diagnosis is denoted by the shaded blue area under the curves.

The temporal appearance of autoantibodies before diagnosis is another parameter that distinguishes autoimmune diseases harboring autoantibodies against intracellular and extracellular autoantigens. These autoantibody studies are based on retrospective, longitudinal serum samples stored in biobank repositories. The first autoimmune disease to be interrogated for prediagnostic autoantibodies was T1D, in which autoantibodies against autoantigens such as insulin, GAD65, and IA2 are present approximately 4–10 years before patients require insulin replacement therapy (55). This lengthy interval of autoantibody seropositivity preceding T1D diagnosis corresponds to chronic subclinical autoimmune attack on the pancreatic beta cells that produce insulin (**Figure 3A**).

T1D is not unique in this respect. Studies of several other autoimmune diseases having mainly intracellular autoantigens also demonstrate a long subclinical phase. In systemic lupus erythematosus, seropositive autoantibodies against Ro52, Ro60, and DNA were detectable on average 10 or more years before diagnosis (56). In rheumatoid arthritis (57, 58), Sjögren’s syndrome (59), and systemic sclerosis (60), autoantibodies to a number of intracellular autoantigens were discovered that antedate the clinical diagnosis. In the case of Sjögren’s syndrome (**Figure 3B**), autoantibodies against Ro52 and Ro60 are often detected in the earliest retrospective serum sample available and can appear up to 18 years before diagnosis (59). Detection of seropositive autoantibodies to intracellular proteins in advance of overt symptoms suggests that an underlying subclinical immune dysfunction may be present long before recognition of the clinical symptoms. It is important to note that for many of these diseases, the presence or extent of early tissue damage is simply unknown because longitudinal tissue biopsies are unavailable.

In contrast to the autoimmune diseases with autoantibodies against intracellular targets, the first detection of seropositive autoantibodies against extracellular autoantigen-driven diseases generally coincides with clinical diagnosis. This is consistent with the principle that these autoantibodies directly cause illness. In anti-GBM autoimmune disease, elevated autoantibodies against the collagen IV-alpha 3 autoantigen are only detectable approximately ≤1 year before diagnosis and not at earlier presymptomatic time points (61). However, in this same study autoantibodies against the intracellular MPO and PR3 were found years before autoimmune kidney disease onset at a average time of 3.25 years consistent with the possibility that they might reflect subclinical immune dysfunction. In membranous nephropathy, another autoimmune kidney disease, longitudinal analysis of future clinical cases showed that 56% of the seropositive patients became seropositive for PLA2R autoantibodies ≤1 year before diagnosis (**Figure 3C**) (62). Another 44% of membranous nephropathy cases showed PLA2R autoantibodies several years before diagnosis, reflecting the relapsing and remitting nature of this autoimmune disease (62). Graves’ disease has provided particularly insightful information because this autoimmune disease shows autoantibodies to both intracellular and extracellular proteins (63). Pre-diagnostic samples from Graves’ disease patients revealed that seropositive autoantibodies against the intracellular thyroid peroxidase were present at a frequency of 31, 49, and 57% at −7 years, −1 year and at the time of diagnosis, respectively (63). In contrast, autoantibodies against the extracellular thyroid-stimulating hormone receptor rose dramatically near the time of clinical presentation and diagnosis. Thyroid-stimulating hormone receptor autoantibody seropositivity was 2% at −7 years, 20% at −1 year, and 55% at the time of diagnosis (**Figure 3D**). These findings in Graves’ disease highlight how autoantibodies against the intracellular protein can circulate for a long time, likely reflecting persistent, low-level autoimmune damage to the thyroid gland. However, the key drivers of productive, symptomatic presentation are the pathogenic autoantibodies that bind to extracellular thyroid-stimulating hormone receptor and thereby activate its signaling.

In summary, autoimmune diseases with autoantibodies against intracellular or extracellular proteins show markedly different patterns of seropositivity during the course of the disease. Autoantibodies to extracellular targets appear close in time to diagnosis because they often cause the autoimmune disease. In contrast, autoimmune diseases harboring autoantibodies to intracellular proteins show detectable humoral responses several years, to even decades, before diagnosis, implying that autoimmune mechanisms are both active and persistent for a sustained period of time. It is possible that some subjects with such a prolonged subclinical phase could experience irreversible tissue damage, and this may in turn impede or prevent effective treatment. Nevertheless, the early warning sign indicated by autoantibody responses against intracellular autoantigens can potentially provide a window of opportunity to thwart the onset of frank autoimmune disease through interventional therapy.

### Mechanistic Differences Imply Different Treatment Modalities for Autoimmune Diseases

Traditionally, autoimmune diseases have been treated with broadly immunosuppressive drugs including steroids, azathioprine, methotrexate, and cyclosporin, which inhibit many different types of immune cells. More recently, targeted therapies such as depletion of specific immune cell subpopulations, anti-cytokine blockade, or inhibition of immune cell signaling pathways are being used to treat autoimmune diseases. In this section, we describe various pathological mechanisms in different autoimmune diseases harboring autoantibodies against intracellular and extracellular proteins and focus on several diseases where this information guides specific treatment strategies.

**Figure 4:**
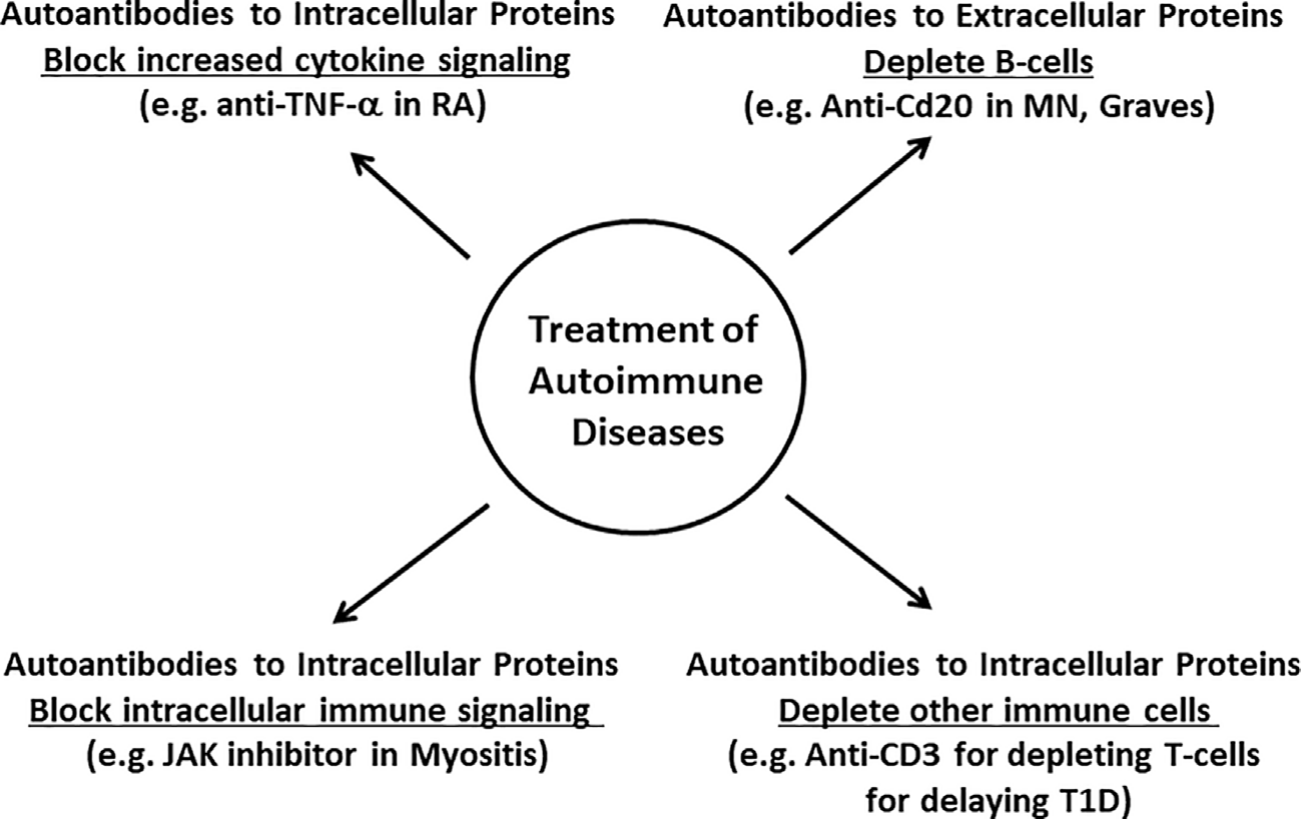
Conceptual foundation for treating autoantibody diseases harboring either autoantibodies to intracellular or extracellular proteins. As shown above, autoimmune diseases with pathogenic autoantibodies to extracellular targets proteins can often be successfully treated with anti-CD20 therapy. In contrast, autoimmune diseases harboring autoantibodies to intracellular proteins require a more tailored approach involving drugs that deplete other immune cell types, block cytokines, or immune signaling pathways.

Autoimmune diseases exhibiting pathogenic autoantibodies against extracellular target proteins often represent ideal cases to employ B lymphocyte-depleting therapies to reduce levels of deleterious autoantibodies. One targeted treatment approach that works well for many autoimmune diseases harboring pathogenic autoantibodies against extracellular targets is rituximab. This anti-CD20 monoclonal antibody therapy eliminates CD20-expressing B lymphocytes, but not plasma cells, stem cells or pro-B-cells. Rituximab acts by causing antibody-dependent complement cytotoxicity and antibody-dependent cell-mediated cytotoxicity, hence causing the death of the CD20-expressing B lymphocyte subpopulation (64, 65). As illustrated in **Figure 4**, rituximab shows efficacy for decreasing autoantibodies to extracellularly located autoantigens in a number of autoimmune diseases including anti-GBM disease (66), myasthenia gravis (67), neuromyelitis optica (68), pemphigus (69), and interferon-gamma autoantibody disease (70). Importantly, in those autoimmune diseases, a significant reduction in pathogenic autoantibodies typically coincides with clinical improvement.

In SLE, rituximab has proven therapeutic benefit and is able to decrease the Systemic Lupus Erythematosus Disease Activity Index (SLEDAI) and significantly lower proteinuria (71). Anti-DNA autoantibodies are a key SLE biomarker, in which anti-DNA autoantibodies enhance cytokine production and can deposit in the kidney to cause pathogenesis (72). The clinical efficacy of rituximab in SLE may be due to its ability to decrease immune complex formation by anti-DNA and other autoantibodies and prevent complement activation, thereby limiting kidney and other tissue damage. In contrast, rituximab and other drugs targeting B-cells often have more variable outcomes when treating other diseases such as Sjögren’s syndrome, or myositis. In Sjögren’s syndrome, B-cell depletion with rituximab provides limited objective improvement of sicca symptoms (73–75). Rituximab shows variable efficacy for dermatomyositis or polymyositis (76). Another B-cell targeting biologic, belimumab, blocks B-cell activating factor, but it does not improve salivary flow or eye inflammation in Sjögren’s syndrome (77). One limitation of using B-cell targeting drugs in diseases with intracellular autoantibodies such as Sjögren’s syndrome is that patients typically seek therapy at late stages, by which time autoantibodies, immune activation, and tissue damage may have persisted for many years.

Many autoimmune disorders with autoantibodies to intracellular autoantigens often involve T-cell-mediated tissue destruction rather that B lymphocyte antibody-mediated damage. In type I diabetes, a large body of evidence suggests that T-cells play a major role in the destruction of pancreatic beta cells, consistent with the finding that T-cell targeted drugs can attenuate beta cell destruction (18). In accordance with these findings is the observation that anti-CD3 antibody therapy with the drug teplizumab, which kills T-cells, delays the progression of type I diabetes in high-risk patients by suppressing CD8+ lymphocytes and thereby blunting the T-cell mediated attack (**Figure 4**) (78).

Another strategy for treating autoimmune diseases is to target T-cell signaling pathways involved in immune activation. One important molecule is the interferon-γ activated Janus kinase signal transducer (JAK) that acts downstream of cytokine signaling to activate STAT transcriptional targets (79). Presently, there are several JAK inhibitors in various phases of development (e.g., tofacitinib, baricitinib, and upadacitinib, figlotinib). The most well-studied among the FDA-approved JAK inhibitors is the orally-active small molecule, tofacitinib, which is therapeutically beneficial for rheumatoid arthritis and psoriatic arthritis (**Figure 4**) (80, 81). In patients with myositis-associated pulmonary disease harboring anti-MDA5 autoantibodies, tofacitinib showed promise by decreasing lung inflammation and improving pulmonary function (82). Many clinical trials with JAK inhibitors are ongoing and this class of drug shows significant promise for treating multiple autoimmune diseases.

Autoimmune diseases with autoantibodies against intracellular target proteins can also involve upregulated cytokine production as a driver of pathology. In several autoimmune diseases, one important treatment strategy is to counteract cytokine-mediated immune activation (**Figure 4**). In rheumatoid arthritis and psoriatic arthritis, elevated levels of the cytokine TNF-alpha1 mediates the inflammation that destroys joints and tissues (83, 84). For treatment of rheumatoid arthritis, several different TNF-alpha inhibitors, such as monoclonal antibody-based therapy (e.g., infliximab, adalimumab, golimumab), or a fusion protein that sequesters TNF-alpha and consists of the extracellular domain of TNF receptor 3 and IgG1-Fc (etanercept) are employed (85). In addition to elevated levels of TNF-alpha, gene expression profiling of SLE, myositis, and systemic sclerosis have identified a type I interferon activation signature (86). In SLE, levels of interferon-alpha cytokine are elevated and correlate with disease flare ups (87). Based on these and other findings, sifalimumab, a monoclonal antibody that binds and blocks interferon-α activity, is efficacious for treating SLE (88), further supporting the key role of interferon-alpha signaling in the pathogenesis of this disorder (**Figure 4**). However, interferon alpha is only one of multiple but related cytokine proteins, and an alternative approach dampens signaling by targeting the common interferon alpha receptor (IFNAR) using the monoclonal antibody drug anifrolumab, which has been shown to reduce symptoms in moderate to severe SLE (89). A recent phase III trial of monthly anifrolumab met its primary endpoint and demonstrated a higher percentage of patients with a beneficial response compared to placebo; additionally, secondary analyses demonstrated decreased glucocorticoid use and reduced severity of skin disease (90).

In summary, autoimmune disease with pathogenic autoantibodies against extracellular targets will often respond to rituximab, if treated early. In contrast, autoimmune diseases characterized by autoantibodies mainly against intracellular proteins are typically driven by T-cells and other immune cells rather than B lymphocytes often do not, or only partially respond to rituximab. Tailored treatments for many of these diseases are less well-developed and involve therapies targeting several different types of immune cells, cytokines, and signaling pathways.

## Conclusions

In this review, we describe how many autoimmune diseases can be segregated based on whether they have autoantibodies mainly against either extracellular or intracellular target proteins. This classification provides insight into mechanisms of autoimmunity, temporal appearance of the autoantibodies and rational foundations for treatment. While most patients with an autoimmune disease generally have one or the other type of autoantibodies exclusively, some patients show more complicated patterns. Some patients who initially feature autoantibodies against intracellular proteins may later acquire pathogenic autoantibodies to extracellular proteins as the disease progresses. This is best documented in Graves’ disease, where autoantibodies against intracellular proteins appear early in the course of disease and autoantibodies against the extracellular thyroid stimulating hormone receptor develop later, coincident with clinical symptoms. As has been described for vasculitis, predominantly intracellular autoantigens such as PR3 and MPO are transiently expressed on the cell surface, and thereby they become accessible to autoantibody binding and are directly involved in disease pathogenesis. There likely are other, yet to be discovered, intracellular proteins that may be recognized by autoantibodies when presented transiently on the cell surface and thus can directly participate in the autoimmune process.

The segregation of autoimmune disease based on intracellular and extracellular autoantigens may also have limitations due to an incomplete assessment of autoantibodies. For example, several studies have shown that rituximab is beneficial in systemic sclerosis (91, 92), a disease classified in this review as having autoantibodies only against intracellular autoantigens. Based on the positive clinical results following rituximab treatment, it is possible that unidentified pathogenic autoantibodies directed against extracellular targets are mediating autoimmunity and abnormal fibrotic matrix accumulation. Alternatively, the positive effects observed with rituximab may involve other functions of B-cells besides antibody production such as antigen presentation or interactions with immune cells.

There remain many unanswered questions about autoantibody production in the context of autoimmunity. For example, little is known about the source of B-cells producing the autoantibodies, whether there are B-cells located in ectopic lymphoid-like structures outside of the spleen, or whether lymph nodes are involved. In the case of Sjögren’s syndrome, within the salivary glands of patients, germinal center-like structures have been found that produce the Ro52, Ro60, and La autoantibodies (93), although less is known about whether ectopic lymphoid-like structures contribute to autoantibody production in other conditions. In addition, new technologies can also be used to analyze autoantibodies, such as mass spectroscopy revealing that in certain autoimmune conditions the presence of public clonotypes of autoantibodies; common antibodies present in different patients (94, 95). This approach could be complemented by the cloning and sequencing of the B-cells producing autoantibodies. One recent study found that rheumatoid factor autoantibodies produced from the B-cells of Sjögren’s syndrome patients had mutations in known B-cell lymphoma driver genes, potentially explaining the clonal expansion of autoantibody producing cells (95). Moreover, sequencing of the immunoglobulins from these cells revealed unique amino acid residues in the rheumatoid factor antibodies that cause insoluble aggregates of immunoglobulins to form, which likely explains why they precipitate from patients’ sera at lower temperatures. Lastly, other technologies such as single-cell RNA sequencing and spatially resolved RNA sequencing could be exploited to characterize and provide new granular insight into the spatio-temporal alterations in immune cell populations and signaling present in the affected tissues of patients in the different autoimmune diseases.

In conclusion, a characterization of autoimmune disorders as we have presented here provides a framework for their mechanistic study and for developing appropriate therapeutic strategies. The observation that autoimmune disease with humoral responses against intracellular proteins have a prolonged seropositive incubation period suggests preemptive screening might identify patients who would benefit from early intervention to reverse or delay the onset of disease. Retrospective studies monitoring the exact temporal appearance of seropositive autoantibodies in parallel with other biomarkers may yield additional insight into potential environmental triggers and other factors that cause or drive disease progression. With regard to disorders caused by pathogenic autoantibodies against extracellular proteins, understanding mechanistic information about why some autoimmune patients show spontaneous remission with the natural disappearance of their autoantibodies may yield new treatment approaches for these diseases. Future strategies will also be developed for treating pathogenic autoantibody diseases by targeting and ablating specific autoantibody-producing B-cells (96), which could provide substantial clinical benefit with fewer off-target side effects.

## Author Contributions

PB initially drafted the review. MI, JK, and BW contributed intellectually through multiple edits, revisions, and refinement of the manuscript. PB and MI drafted the figures. All authors contributed to the article and approved the submitted version.

## Funding

This work was supported by the Intramural Research Program of the National Institute of Dental and Craniofacial Research and the Clinical Center, NIH. The content of this publication does not necessarily reflect the views or policies of the Department of Health and Human Services, nor does mention of trade names, commercial products, or organizations imply endorsement by the U.S. Government.

## Conflict of Interest

The authors declare that the research was conducted in the absence of any commercial or financial relationships that could be construed as a potential conflict of interest.
